# Time-series analysis of transcriptomic changes due to permethrin exposure reveals that *Aedes aegypti* undergoes detoxification metabolism over 24 h

**DOI:** 10.1038/s41598-023-43676-9

**Published:** 2023-10-02

**Authors:** Lindsey K. Mack, Geoffrey M. Attardo

**Affiliations:** Department of Entomology and Nematology, University of California, Davis, Davis, CA USA

**Keywords:** RNA sequencing, Transcriptomics

## Abstract

Insecticide resistance is a multifaceted response and an issue across taxa. *Aedes aegypti,* the mosquito that vectors Zika, dengue, chikungunya, and yellow fever, demonstrates high levels of pyrethroid resistance across the globe, presenting a challenge to public health officials. To examine the transcriptomic shifts across time after exposure to permethrin, a 3’Tag-Seq analysis was employed on samples 6, 10, and 24 h after exposure along with controls. Differential expression analysis revealed significant shifts in detoxifying enzymes and various energy-producing metabolic processes. These findings indicate significant alterations in gene expression associated with key energy mobilization pathways within the system. These changes encompass a coordinated response involving lipolysis, beta-oxidation, and the citric acid cycle, required for the production of energetic molecules such as ATP, NADH, NADPH, and FADH. These findings highlight a complex interplay of metabolic processes that may have broader implications for understanding insect physiology and response to environmental stimuli. Among the upregulated detoxifying enzymes are cytochrome P450s, glutathione s-transferases and peroxidases, and ATP-binding cassette transporters. Additionally, eight heat shock genes or genes with heat shock domains exhibit the highest fold change across time. Twenty-four hours after exposure, samples indicate a global downregulation of these processes, though principal component analysis suggests lasting signatures of the response. Understanding the recovery response to insecticide exposure provides information on possible new genetic and synergist targets to explore.

## Introduction

Global insecticide intolerance is an issue across taxa. Insects overcome this stressor via a combination of genetic resistance, metabolic resistance, and behavioral resistance^1^. Due to the multifaceted nature of this response, achieving long-lasting population control with current insecticides is difficult, if not impossible^2^. Further exploration into this response could yield important results for the development of new insecticides with alternate modes of action or synergists to enhance efficacy of existing insecticides.

A particularly important model for exploring this complex response is the yellow fever mosquito, *Aedes aegypti*, due to ease of collection and rearing, as well as real world implications of this insect in public health. In addition, the availability of an array of genetic tools for this species facilitate in depth investigation and manipulation for research and control purposes. *Ae. aegypti* threatens over half the world’s population with one or more of the viral pathogens it carries (Zika, dengue, chikungunya, yellow fever, etc.), and presents a significant burden on the global health system. This mosquito is found on all continents aside from Antarctica due to physiological characteristics that improve its invasion abilities. *Ae. aegypti* lay eggs which are desiccation resistant, surviving for months after oviposition. Typically, eggs are laid in man-made containers and humans are the preferred host of these mosquitoes. In 2013, *Ae. aegypti* first established a population in California’s Central Valley, but has now spread as far south as the Mexico border and as far north as Redding, CA^3^. Unfortunately, *Ae. aegypti* poses a significant challenge to public health officials due to widespread insecticide resistance.

Pyrethroids are a commonly used class of insecticides in public health applications as they show significant toxicity in arthropods, but relatively little toxicity to mammals due to differences in the target site binding affinity and metabolic rate^4^. These chrysanthemum derived xenobiotics bind to open voltage gated sodium channels, causing these channels to remain open leading to constant depolarization of the membrane leading to death in insects^5^.

In *Ae. aegypti,* several single nucleotide polymorphisms (SNPs) in the voltage gated sodium channel gene (VGSC) are recognized as conferring resistance, though the prevalence of these SNPs is location dependent. In California, the primary SNPs are V1016I, F1534C, and V410L, though the frequency of these differs across the state^6^. Additionally, overexpression or overactivation of detoxifying genes are associated with resistance, though in *Ae. aegypti* there are over 150 genes that code for these types of enzymes and the overexpression and activation of these genes in resistant mosquitoes is highly variable geographically^7^.

Gene expression responses are not instantaneous but rather complex and time intensive. In the xenobiotic response, there are multiple stages of detoxification. Stage I is dominated by cytochrome p450s (CYPs), initially neutralizing xenobiotics and tagging them for further breakdown. Stage II is dominated by glutathione-s-transferases (GSTs) which breakdown these molecules further into compounds that can be excreted. Stage III is characterized by various transporter proteins moving these compounds across the membrane into the gut or rectum for excretion^8^.

While many genes have been identified as conferring resistance in *Ae. aegypti*, the temporal response of the transcriptome to exposure to these compounds is relatively unknown, especially in adults. Researchers studied the response to pyrethroid exposure in *Anopheles funestus* larvae across time, but application of pyrethroids typically target adult mosquitoes rather than the aquatic larvae^9^. Defining this response over time is important for a nuanced understanding of the progression and complexities of the response to pyrethroid exposure across multiple physiological systems. Additionally, a deeper understanding of this response could highlight genes or pathways that may be ideal targets for novel monitoring and/or control methods, both chemical and genetic. To study this response in adult *Aedes aegypti*, a high-throughput analysis of gene expression over time was performed using 3’Tag-seq. Here, we characterize the response of a field strain of *Aedes aegypti* to a sub-lethal pyrethroid challenge over the course of 24 h. The results reveal the identity and timing of insecticide responsive genes and pathways involved in the xenobiotic response of this strain of *Ae. aegypti.*

## Results and discussion

### Principal component analysis highlights major divergence in 24-h post-permethrin treatment expression profile

**Figure 1:**
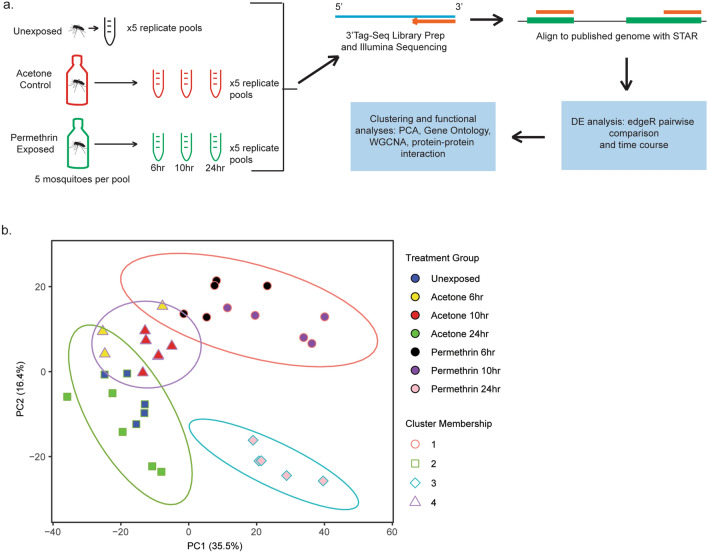
Summary of experimental design and cluster analysis of expression profiles. (**a**) Depiction of experimental design and (**b**) Principal component analysis of all samples using log transformed gene counts. Kmeans clustering analysis indicated 4 clusters to be ideal for the amount of variation in this dataset. Biplot available in Supp. Fig. 1.

To assess transcriptomic changes across the first 24 h after exposure to permethrin, 35 libraries were created from samples collected before exposure, and 6, 10, and 24 h after exposure (summarized in Fig. 1a). These time points were chosen based on a microarray study in *Anopheles gambiae* that showed significant changes in response at these intervals^10^. All 35 libraries produced high quality 3’Tag-Seq single end reads, with an average library size of 4,092,249 reads (min:3,493,811—unexposed replicate 2, max: 4,640,468—24 h post permethrin exposure replicate 3) providing an average coverage of 206 reads per gene of the 19,804 annotated genes in the *Aedes aegypti* genome. 3′Tag-Seq, focuses exclusively on the 3′ end of the transcript. This allows for more specific and consistent read mapping and reduces the number of reads required for robust detection of differential expression (~ 5 million reads in 3′Tag-Seq versus ~ 30 million in standard RNA-seq)^11^. Across samples, 96.7% of reads mapped to the reference genome at least once on average. Gene annotations were extracted from Vectorbase and genes with an “unspecified product” annotation were searched with BlastP against a custom database of the Culicidae and Drosophila databases to search for orthologs of unannotated genes. To filter genes demonstrating low expression levels, annotated genes were required to have a minimum of 1 count per million (CPM) in at least two of the sample replicates. After filtration, 11,155 (56.3%) genes remained which were utilized in downstream analyses.

To assess how samples grouped based on overall expression profile, a principal component analysis was performed to ensure grouping was consistent with treatment conditions. PC1 explained 35.4% of the variation, while PC2 explained 16.4% of the variation (Fig. 1b). Because only 51.8% of the variation in the dataset was explained by PC1 and PC2, further clustering analyses were performed.

To further define the differences and similarities between groups, a kmeans cluster analysis was performed and the gap statistic was calculated to determine the optimal number of clusters within the data set^12^. Through this analysis, it was determined that the data could be categorized into 4 clusters as follows: baseline and 24 h post-acetone treatment, 6- and 10-h post-acetone treatment, 6- and 10-h post permethrin treatment, and 24 h post permethrin treatment (Fig. 1b). Based on the clustering, the acetone exposed controls appear to return to baseline 24 h after treatment, indicating that the use of this reagent as a control in insecticide resistance testing experiments is adequate. To maintain clarity when discussing differences between groups, control samples will be discussed as “acetone treatment” to differentiate from the untreated baseline samples. These samples primarily represent handling controls as acetone should be fully evaporated at time of bottle testing.

Because replicate samples from the 24 h post-permethrin treatment demonstrate the greatest variance relative to the other treatment groups, we further investigated the genes driving this separation using a biplot. Biplot analysis (Supp. Fig. 1), reveals that the gene most strongly influencing this treatment group is *catalase* (AAEL013407), which codes for a protein that protects against oxidative stress by catalyzing the breakdown of hydrogen peroxide into two water molecules. Catalase has undergone thorough investigation in mosquitoes and other flies and is associated with multiple physiological processes including longevity^13^, insecticide resistance^14^, fecundity^15^, pathogen infection^16^, and maintenance of homeostasis after blood feeding^17^. All of these processes play some role in a mosquito’s ability to transmit a pathogen. Further study of the role of catalase in insecticide response, particularly whether the observed increase remains past the 24-h post-exposure mark is important for understanding the intersection of sublethal insecticide exposure and pathogen transmission. For this reason, exploring chemical agents that exacerbate ROS production or disrupt catalase activity could be potential synergists for current mosquito control chemical agents.

### Redox homeostasis processes exhibit significant expression changes over time after insecticide exposure

We next characterized the time course expression response to pyrethroid exposure to holistically assess differential expression. This analysis provides information on the relationship between time points and expression values, and assess which genes are experiencing significant changes in expression across time. To accomplish this, we fit a cubic regression spline curve with 3 degrees of freedom to model expression trends across the time course and used edgeR^18^ to assess the statistically significant differences between permethrin and acetone exposed groups. The analysis revealed significant responses over time after insecticide exposure with 383 genes upregulated and 200 downregulated.

**Figure 2:**
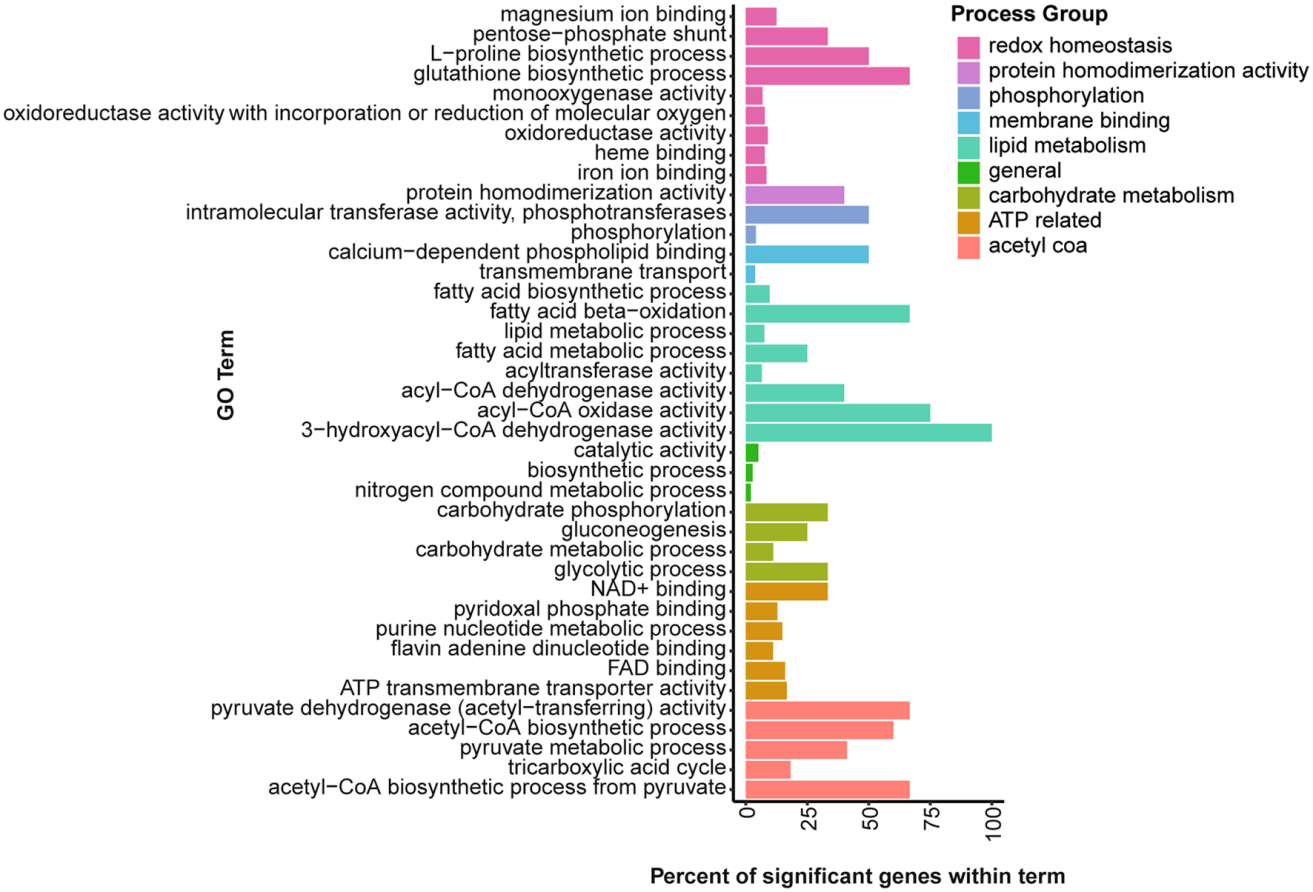
Gene ontology analysis of genes upregulated throughout the time course. Time course modeled using cubic regression spline curve and pairwise comparison between treatments. A high percent of significant genes within a term indicates a large proportion of genes annotated with a specific term were found significantly upregulated in this analysis.

We performed a gene ontology (GO) analysis on the upregulated and downregulated genes to identify enriched functions within these groups. Of the 383 upregulated genes, we found enrichment of 5 primary GO categories. Among these are acetyl-CoA biosynthesis, glucose metabolism, lipid metabolism, redox homeostasis processes, and ATP production (Fig. 2).

Redox homeostasis processes are the primary response component to many stressors, including insecticides. Pyrethroid exposure causes oxidative stress, resulting from an imbalance of reactive oxygen species (ROS) and enzymes capable of neutralizing them^19–22^. Free radicals are formed by many cellular processes including the electron transport chain, fatty acid beta oxidation, and many other processes that require the breaking of molecular bonds such as the breakdown of xenobiotics. Gene families involved in the neutralization of free radicals to maintain redox homeostasis are also involved in the breakdown of xenobiotics, these include CYPs and GSTs^20,23^. Expression of these genes is often induced by increases in ROS^24^. ROS can cause conformational changes that result in the release of a cofactor from a transcription factor allowing for nuclear localization of said transcription factor. One example is the release of the KEAP-1 protein from cap n collar transcription factor^25^.

The *cap n’collar C isoform* (*CncC*) (AAEL019563 in *Ae. aegypti)* is the invertebrate equivalent of the vertebrate nuclear factor erythroid 2-related factor (*Nrf2*), which is known to bind antioxidant response elements^26^. *CncC* binding to these promoters leads to the expression of the previously mentioned gene families under insecticide stress^27^. Importantly, *CncC* is constitutively active in resistant populations of *Drosophila* and *Anopheles gambiae*^28,29^*.* Studies have also found that this transcription factor is responsible for metabolic shifts in response to stress, which are illustrated by this data set as well^30,31^. Interestingly, in this dataset, AAEL019653 does not display a significant change until 24 h after exposure, when it is downregulated (logFC =  − 1.04, FDR = 0.00019). It is unsurprising that this gene does not experience any upregulation throughout the time course as it is a constitutively expressed transcription factor which is released from its repressor upon increases in ROS within the cell. Downregulation of CncC at 24 h suggests a reduced demand for protein. Given that this protein is regulated at the post-translational level it is unknown whether the reduced transcript abundance is an attempt to lower the levels of CncC protein to reduce regulatory activity or if it is a response to a reduction in CncC turnover thereby reducing demand for new protein synthesis to maintain equilibrium.

Genes associated with both lipid and carbohydrate metabolism were upregulated, along with acetyl-CoA biosynthesis. This is consistent with results in other studies on the response to insecticide exposure^10,21,32^. Acetyl-CoA is derived from the catabolism of proteins, lipids, and carbohydrates. Acetyl-CoA is a primary monitor of the metabolic state of an organism. When acetyl-CoA is plentiful, it is shuttled into lipid synthesis and while in a depleted state, it is transported into the mitochondria for ATP synthesis^34^. Other processes involved in ATP production include genes involved in purine synthesis, NAD binding and FAD processes. These processes generate high energy electron acceptors in the electron transport chain. This paired with ATP transmembrane transport overrepresentation clarifies the energetic stress the insect is under after exposure to a xenobiotic. Additional evidence supports the role of *CncC* in increased lipid and carbohydrate metabolism, as constitutive activation of this protein leads to a decrease in lipid stores in *Drosophila* and mouse cells showed increased glucose uptake in the same condition^31,33^*.*

While compelling, the associations of CncC's role in mediating the array of responses to pyrethroid exposure remain speculative. Additional work is needed to define the role of CncC in the resistance phenotype. Specifically, there is a need to further investigate the differences in gene expression, protein localization, and DNA binding dynamics of CncC between susceptible and resistant strains sharing a genetic background, in the context of insecticide exposure.

### Heat shock proteins exhibit the largest fold changes among upregulated genes across time

**Figure 3:**
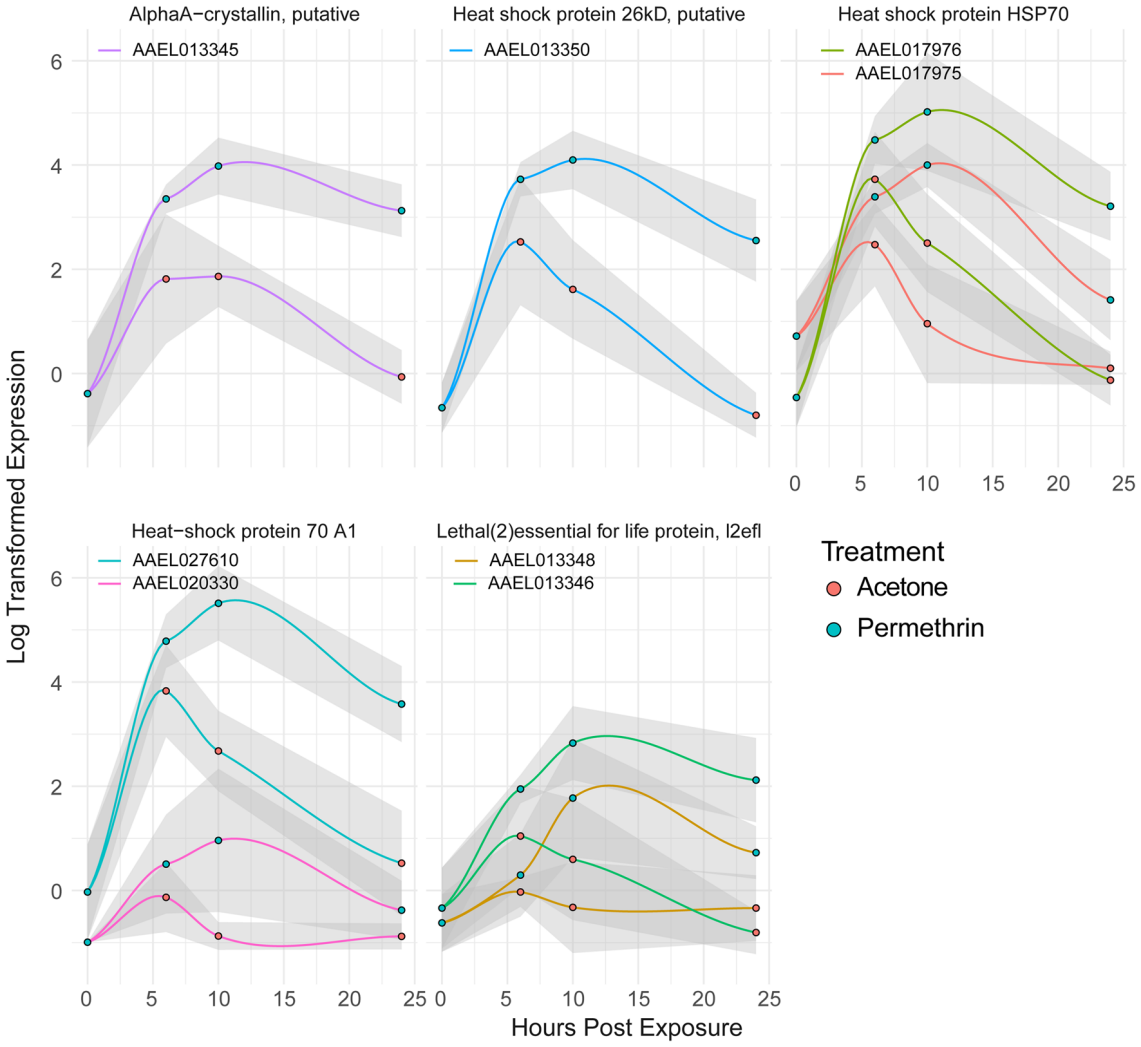
Heat shock proteins experience large fold changes over time after exposure to permethrin. Genes are faceted by product descriptions found in Vectorbase, and 95% CI shown with grey shading. *AlphaA-crystallin* is a small HSP (Jakob et al. 1993). *l2efl* is a member of the HSP20 family (Runtuwene et al. 2020).

Eight of the top 20 genes with the largest fold change belong to the heat shock protein (HSP) family (Fig. 3). Each of the HSPs displays a similar patterns of transcript abundance over time, with an increase through 10 h followed by a gradual decline. Acetone treated samples show a response in these gene as well, though expression levels return to baseline at 24 h while permethrin treated samples still have elevated expression. HSPs are associated with response to environmental stressors and have been associated with insecticide exposure response in many insects^35–40^. HSPs have a wide array of functions, aiding in all aspects of protein processing while not acting as a part of the mature protein^41^. One of these functions is as chaperones to aid in initial protein folding or to repair damage following stress. The role these molecules play in insecticide response may be due to protein damage due to ROS. One study found protein oxidation products increase following inoculation with the larvicidal bacteria *Bacillus thuringiensis kurstaki,* however, direct evidence of these products has not been studied in adult mosquitoes following insecticide exposure^42^.

In a warming climate, the interactions between heat stress and insecticide resistance have the potential to pose major issues for vector control professionals^43^. There is even evidence that heat tolerance can protect from insecticide exposure and vice versa^35,40^. Continued research on the development of cross-resistance between heat and pyrethroids will be important for future vector control efforts.

### Time point specific pairwise comparisons: seven detoxifying genes upregulated at all time points

**Figure 4:**
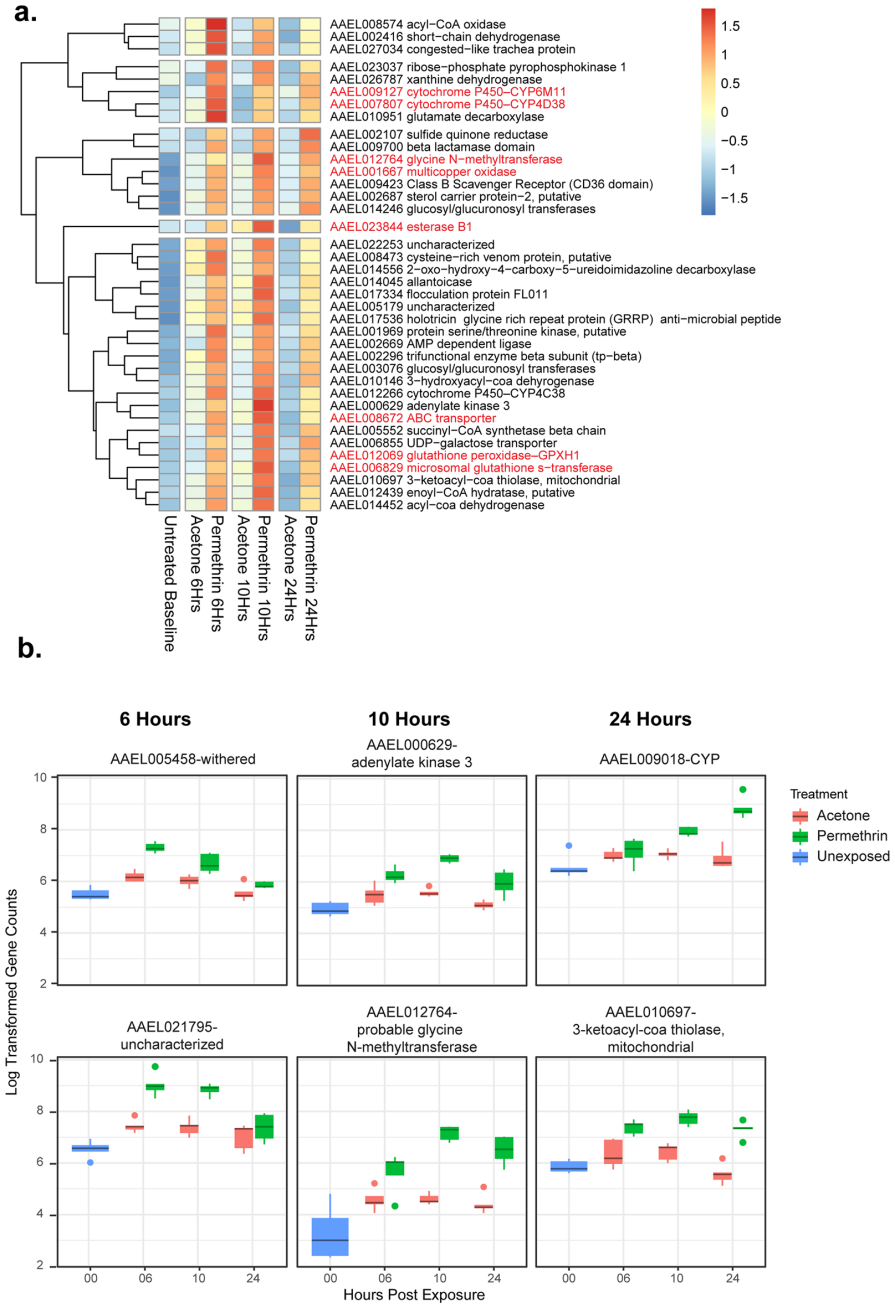
Summary of pairwise comparisons. (**a**) Heat map of genes upregulated at all time points (FDR < 0.05), red text indicates detoxifying genes. (**b**) Top 2 upregulated genes with smallest FDR at each time point.

To investigate time point specific detoxification signatures, we employed pairwise comparisons at each time point in edgeR^18^. 1245 (11.16%) were differentially expressed in at least 1 time point, 645 upregulated and 599 downregulated, using an FDR cut off of 0.05. Thirty-eight of the 645 upregulated genes were upregulated at all 3 time points (5.9%) while only 1 gene (a putative oxidase/peroxidase) was downregulated at all 3 time points (AAEL019639, FDR = 7.97 × 10^−9^) (Fig. 4b).

Gene ontology analysis was used to explore functional characteristics of genes upregulated at all time points. Due to the small number of genes, the classic fisher algorithm was used which tests GO terms independently and is therefore less conservative than the TopGO default algorithm. Genes shared across time points fall into four primary categories: lipid metabolism, detoxifying enzymes, purine breakdown, and FAD binding (Fig. 4a). Among the detoxifying enzymes are *CYP6M11, CYP4D38, esterase B1, CYP4C38, GPHX1*, a microsomal GST and an ABC transporter. These are part of different stages of detoxification. It could be speculated that these detoxifying enzymes exhibit a more generalized function, aiding in the breakdown and excretion of various byproducts. Alternatively, the process of insecticide detoxification in insects might be so slow that they continue to sequester insecticides and break down entire molecules, even 24 h after exposure. This could be an important consideration for vector control professionals who routinely perform insecticide resistance testing in the US where the CDC bottle bioassay is common, as the time at which they are observing knockdown may not be an accurate depiction of resistance state^44^.

### Fatty acid beta oxidation and ATP production are predominant processes at 6-h post-exposure

Based on the time point specific differential expression analysis, six hours post exposure 95 genes were upregulated while 15 genes were downregulated. Processes overrepresented at this time point are related to fatty acid beta oxidation and ATP production and transport. The most significantly upregulated gene is that coding for the protein carnitine O-palmitoyl transferase (*whd*) (AAEL005458, FDR = 2.36 × 10^−5^) which is involved in fatty acid beta oxidation^45^. Of the 95 upregulated genes, 34 (35.7%) are only upregulated at 6 h, one of which is the transcription factor, *Hnf4* (AAEL011323, FDR = 0.028). This factor is associated with a metabolic switch to oxidative phosphorylation in *Drosophila* and fatty acid beta oxidation in *Ae. aegypti*^46,47^*.* Additionally, it is likely that *whd* is activated by *Hnf4,* as researchers found that the expression of *whd* significantly decreased upon RNAi knockdown of *Hnf4*^47^*.*

Downregulated genes were more specific to the 6-h time point, with 12 of the 15 only showing reduced abundance at 6 h. However, there was no significant overrepresentation of function among this group based on a gene ontology analysis. An interesting gene within this group is a cytochrome P450, *CYP9J22* (AAEL014619, FDR = 0.017), as the CYP9J family is implicated in insecticide detoxification. Because it is downregulated so early in the time course, it may be one of the first CYPs to respond to permethrin followed by a drastic downregulation, or it may just be an example of strain specific detoxifying gene responses^7^. The 6-h time point shared 15 upregulated genes with the 10-h time point, though this group did not contain any significantly overrepresented GO terms. However, among these genes is *whd,* indicating a continuation of increased fatty acid beta oxidation into 10 h post exposure.

### Carbohydrate metabolism elevates ten hours post-exposure and sustains through 24 h

Gene expression changes at 10-h post-exposure represent a switch in energy source as displayed in the time point specific differential expression analysis, as well as the peak of expression for genes displaying differential upregulation at all time points. At ten hours post exposure, 219 genes were upregulated, while 77 genes were downregulated. GO term overrepresentation among upregulated genes shows an increase in carbohydrate metabolism, with a continuation of fatty acid beta oxidation as well. This may indicate that mosquitoes have burned through much of their lipid stores by 10 h post exposure, leaving them to function off of carbohydrates derived from sugar feeding. The most significant upregulated gene is that coding for glycine N-methyltransferase (*GNMT)* (AAEL012764, FDR = 6.31 × 10^−7^). This gene has not been previously associated with insecticide response to our knowledge, however the role of the mouse ortholog in stress response has been investigated. Knockout of *GNMT* in mice resulted in a reduction in expression of detoxifying genes including CYPs, GSTs, catalase, and superoxide dismutase, as well as an increase in lipid peroxidation products^48^. Further study of the role of this gene in insecticide response may provide additional information on resistance mechanisms.

Eighty-one (37.0%) upregulated genes are only upregulated at 10 h, though no significant GO term overrepresentation was found. Among these are 4 detoxifying genes previously associated with insecticide exposure: *GSTD6*, *CYP9J9*, *CYP304B2*, and *CYP304C1*, and 1 gene previously studied in response to insecticide exposure with negative results: *GSTT1*^27,49–52^. Differing results in our study may be due to observation of expression changes over a longer span of time or merely strain specific differences. Forty-five of the 77 (58.4%) downregulated genes were only downregulated at 10 h. *GSTT4*, a detoxifying gene previously linked to pyrethroid exposure, is among this group. Although it was found to be overexpressed in a study where *GSTT1* was deemed insignificant^52^. This suggests that the role of individual GSTs in the detoxification response may vary between strains in their response to pyrethroid exposure.

Ten hours post exposure shares 85 upregulated genes with 24 h post exposure, many of which are involved in oxidoreductase activity (among which are *catalase*, *CYP9J6*, *CYP6BB2*, and a CYP with no family assigned), carbohydrate metabolism, and organic acid metabolism. *CYP9J6* and *CYP6BB2* are well associated with pyrethroid response^49,53,54^. In some strains of *Ae. aegypti, CYP6BB2* is increased in copy number*,* and has been specifically shown to aid in metabolism of permethrin^55,56^*.* Additionally, 9 HSP genes or genes with HSP-like binding regions are upregulated, consistent with results from the overall time course differential expression analysis.

### Downregulated genes at 24 h post-exposure are tied to energy consumption

Expression signatures 24-h post-exposure were investigated to further assess broad differences creating the clustering divide observed in Fig. 1b. Twenty-four hours post exposure, 371 genes are upregulated while 476 genes are downregulated. The most significant upregulated gene was a cytochrome P450, possibly part of the CYP6B family, and experienced a twofold change (AAEL009018, FDR = 1.42 × 10^−7^)^57^. This gene has been associated with both pyrethroid and bendiocarb resistance^57,58^. The specificity of its differential expression solely at 24 h after exposure to permethrin in this strain is unknown.

**Figure 5:**
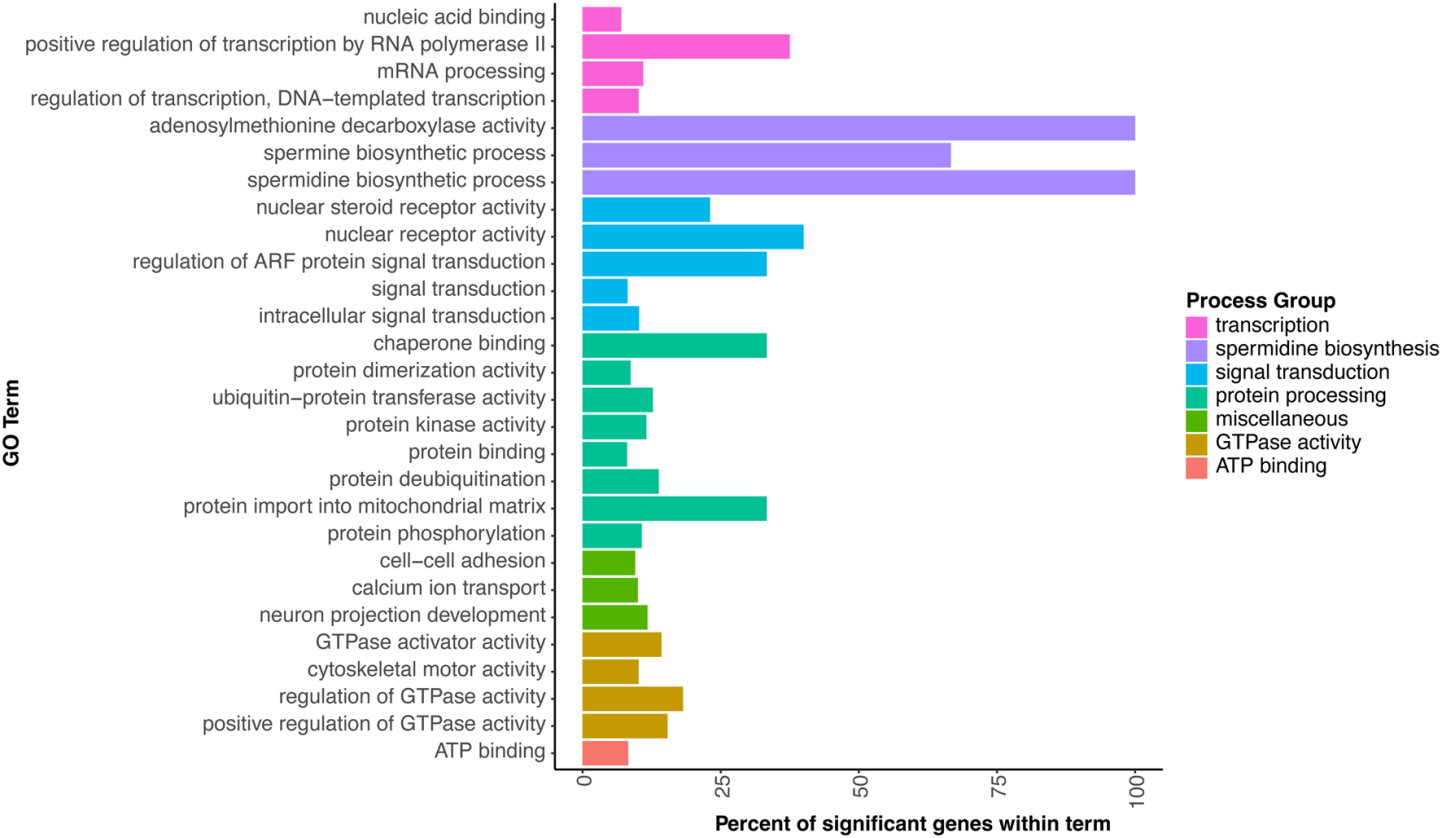
Gene Ontology analysis of downregulated genes at 24 h post exposure. Genes with FDR < 0.05 and logFC < 0 included in analysis.

The upregulated genes at this time point largely mirror the upregulated genes throughout the time course, however the downregulated genes are unique to 24 h, with 445 of the 476 downregulated genes only downregulated at 24 h. The GO hits fall within 6 categories: ATP binding, GTPase activity, protein processing, signal transduction, spermidine biosynthesis, and transcription (Fig. 5). Spermidine is associated with protection against the toxic effects of pyrethroids in zebrafish, though the downregulation of genes involved in its synthesis may indicate that the insect is no longer experiencing toxic effects at this time^59^.

This pattern of activity could be indicative of the insect adjusting its physiological response, possibly moving towards a basal state. The observed decreases in ATP binding, signal transduction, protein processing, GTPase activity, and transcription may suggest a decrease in the molecular responses to pyrethroid exposure. However, it is important to note that our interpretation of these findings is speculative and requires further investigation. The precise mechanisms underlying these changes remain unclear, and additional research is needed to validate this hypothesis and fully understand the insect’s response to pyrethroid exposure.

### Thirty-eight detoxifying enzymes exhibit an association with pyrethroid response

**Figure 6:**
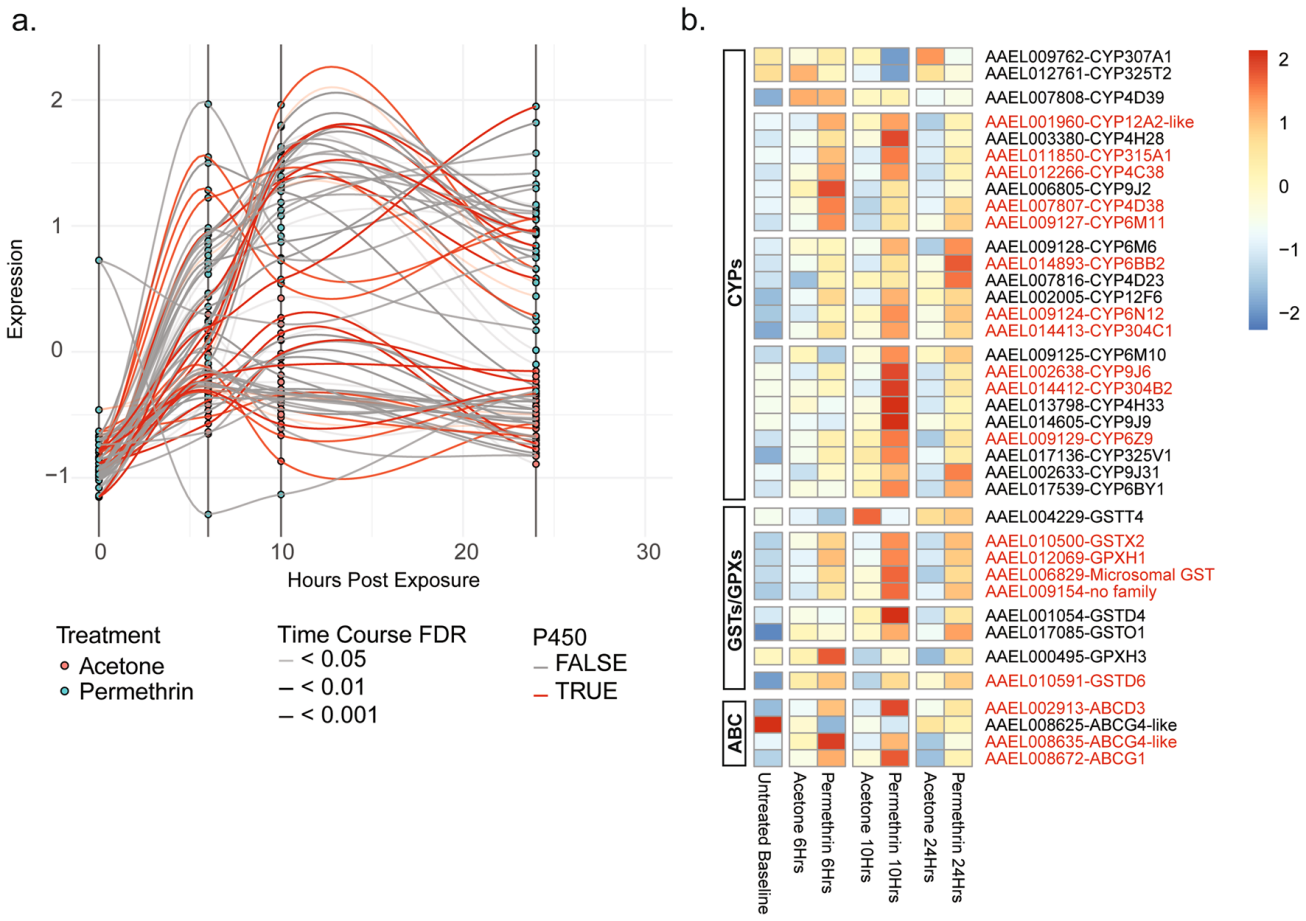
Summary of oxidoreductase activity genes in the WGCNA module with the highest correlation to treatment. (**a)** Line plot of oxidoreductase activity gene expression over time highlighting the domination of CYPs within this group. (**b**) Heat map of CYPs, GSTs/GPXs, and ABC transporters within this module, regardless of FDR. Red text indicates an FDR < 0.05.

To further classify those xenobiotic response-associated genes that may not have exhibited a statistically significant change in expression, we conducted a weighted gene correlation network analysis utilizing the R package WGCNA^60^. This analysis groups genes based on expression patterns over time as well as by their correlation with treatment. Thirty modules were identified with one highly correlated with treatment (MEbrown, Supp. Fig. 3). This module contains 931 genes, 305 of which are differentially expressed using an FDR cutoff of 0.05 from the time course differential expression analysis (245 upregulated, 60 downregulated). Interestingly, no correlation with time was observed in this module. This suggests that the expression changes witnessed over this time course are attributed to the xenobiotic response, rather than merely to time-associated expression alterations. Forty-two genes within the oxidoreductase GO term are significant based on the time course analysis within this group (Fig. 6a). Oxidoreductase activity includes many enzymes associated with detoxification functions.

Within the module most related to treatment are 25 cytochrome P450s (without filtering based on FDR). Eleven of these are significant when considering expression over time while 17 are significant in at least 1 time point (Fig. 6b). Expression patterns of these genes are inconsistent, but cluster into 5 groups based on these patterns. One CYP is significantly downregulated at 10- and 24-h post exposure, *CYP307A1*. This gene is included in the Halloween genes and is the ortholog of the *Drosophila* gene *Spook*. Spook is part of ecdysone synthesis, so this downregulation may have implications for the role of ecdysone metabolism in the insecticide stress response^61,62^.

We also found members of the CYP9J family which is associated with pyrethroid resistance^7,63–66^. Four genes from this family are present in the treatment associated module, 3 of which cluster together based on expression pattern (*CYP9J6, CYP9J9*, and *CYP9J31*). In prior work, researchers performed an extensive micro-array study on rhythmic gene expression in female *Ae. aegypti* heads with a special focus on the CYP9J family and found two groups of co-oscillating members of this family^67^. However, none of the genes explored in that study were differentially expressed in this study.

Nine GSTs/GPXs are present in the treatment-associated module, 5 of which are significant. Expression trends are more consistent within this group, with expression peaking at 10 h post exposure (Fig. 6b). *GSTX2* is associated with DDT resistance in South America, however researchers in Thailand performed in-vitro experiments with this protein derived from a Thai population and found no evidence of direct metabolism of DDT by this enzyme^68,69^. *GPXH1* appears to be induced by xenobiotic exposure, however, overexpression of this gene is not associated with resistance^70^.

Four ABC transporters are in this group, 3 of which are upregulated. ABC transporters are responsible for transporting insecticide and byproducts of insecticide metabolism out of the cell to avoid accumulation of these toxic compounds^71^. Transcription of these are also initiated by *CncC*, and their role in xenobiotic detoxification has been validated by RNAi experiments in crop pests^72^.

### Results are relatively consistent with similar studies across the family *Culicidae*

Insects evolve rapidly as a response to their environment. For this reason, the specific genetic response to insecticide exposure can vary greatly geographically, as well as in closely related species. In a similar time-course analysis (though including time points through 72 h after exposure) of sublethal insecticide exposure response in *Anopheles coluzzii*, authors found oxidoreductase and fatty acid degradation related genes to only increase in expression after 24 h post exposure. This differs from our findings, as we saw immediate increases in expression of genes related to these processes. Additionally, the authors of this study identified changes in gene expression related to DNA repair, which we did not observe. These authors observed an overall downregulation of mitochondrial respiratory process, much different from our observation of overall upregulation of these processes. They hypothesized that this downregulation was likely a mitigator of total ROS load, as the xenobiotic response produces ROS^73^. Further investigation to compare energy production after exposure to insecticide may give interesting insights into variations in the evolution of resistance in different species or to fitness costs related to some components of the insecticide response.

Similarly, the authors also identified many of the same families of CYPs and GSTs showing a significant response to deltamethrin, another pyrethroid insecticide. However, the specific responsive genes were not orthologous between the two species. Specifically, many CYP6M genes responded, though none of these were orthologs of genes detected in our study (Fig. 6)^73^. Additionally, the strain used for these experiments is known to have an increase in constitutive alpha-crystallins expression, one of which we found to have a robust response to permethrin in our study (Fig. 3)^74^.

The environment in which adult and larval mosquitoes inhabit is vastly different, though the chemical challenges each face can be similar. In a study of *Anopheles stephensi* larvae exposed to permethrin for up to 48 h, many xenobiotic detoxification genes and redox balancing genes showed varying responses to exposure. Of particular interest is downregulation of the majority of the heat shock protein genes identified within their dataset^9^. This is much different from our findings, as we observed heat shock protein genes to have some of the highest log fold changes across time (Fig. 3). Additionally, authors did not find a significant upregulation of any GSTs within their data. They did find some CYPs upregulated, though none were orthologous to those in our results (Fig. 6)^9^.

### Differential expression analysis reveals gene candidate for further study

A surprising gene displaying significant upregulation at 6- and 10-h post exposure is a Niemann-Pick type C-2 protein gene (NPC2) (supplementary file 1). This family of proteins is known to act as cholesterol transporters in vertebrates but has a wider array of functions in insects^75^. Insects have duplicated these genes likely in response to environmental pressure, while vertebrate species tend to have one^76^. In mosquitoes, these types of proteins are associated with protection from midgut infection of dengue virus^77,78^. It has yet to be associated with insecticide response. NPC2 is found in lysosomes, which could be acting in insecticide breakdown, though they are known to breakdown biological material (cell components, bacteria, etc.) rather than chemicals. Lysosome function in insecticide metabolism should be explored further to improve our understanding of tolerance and the intersection of insecticide resistance and pathogen transmission.

## Conclusion

Exposure to permethrin results in not only an immediate physiological response, but a response that continues through at least 24 h after exposure. This response is characterized by shifts in metabolism and energy production along with redox balancing and detoxifying genes. Understanding the recovery response to insecticide exposure provides information on possible new genetic and synergist targets to explore. Additionally, observing these genetic trends provides evidence that treating with insecticide at two time points within the same 24 h time period may be effective, though the logistics of such an application would be difficult and likely cost prohibitive. This study also highlights strain specific differences in the response of detoxifying enzymes. Further investigation into the evolutionary mechanisms behind these differences may give improved understanding of the evolution of insecticide resistance.

## Methods

### Mosquitoes

Mosquitoes were collected from Reedley, CA (36.5809032, − 119.4553858,16.51), a small town in the Central Valley of California, USA. This region is known for a wide array of agricultural operations. Collections were completed via oviposition cups. Mosquitoes were reared according to existing protocols for 2 generations in the lab^79^. Briefly, larvae were reared in trays of 200 in 1 L of dechlorinated tap water and fed Fluval fish pellets. Upon pupation, pupae were removed from larval trays and placed in BugDorm #6 cages and fed 10% sucrose ad libitum until the 5th day post-eclosion. To determine the resistance phenotype of this strain to permethrin, a CDC bottle bioassay was performed, using the CDC-recommended diagnostic dose of 43ug/mL^44^. Based on the diagnostic time of 10 min, the strain was determined to be resistant to permethrin (Supp. Fig. 4). Additionally, this strain is homozygous resistant for F1534C, V410L, and V1016I (unpublished data).

### Permethrin exposure

In groups of 15–25, females 5 days-post eclosion were placed in 250 mL Wheaton bottles coated in permethrin at a concentration of 15 ug/mL in acetone or 1 mL acetone as a control for 1 h. Mosquitoes were then removed from bottles and placed in BugDorm #6 cages with access to 10% sucrose water until collection time. Five biological replicates of pools of 5 mosquitoes were collected 6, 10, and 24 h after removal from exposure bottles and homogenized in the lysis buffer supplied with the Zymo Quick RNA Mini-Prep kit (R1054). Additionally, 5 biological replicates of pools of 5 mosquitoes were collected directly from rearing cages prior to permethrin/acetone exposure to act as a baseline. In all, 35 samples were collected, 3 treatment groups were used (baseline, acetone control, and permethrin), and 3 time points were used (6, 10, and 24 h after removal from treatment bottles). RNA was extracted and samples were assessed for contamination with a NanoDrop One^c^, then analyzed via Bioanalyzer for RIN score and concentration by the UCDGC.

### Library prep and sequencing

RNA was submitted to the UC Davis Genome Center for library prep and 3′ Tag-seq analysis. Gene expression profiling was carried out using a 3′-Tag-RNA-Seq protocol. Barcoded sequencing libraries were prepared using the QuantSeq FWD kit (Lexogen, Vienna, Austria) for multiplexed sequencing according to the recommendations of the manufacturer using both the UDI-adapter and UMI Second-Strand Synthesis modules (Lexogen). The fragment size distribution of the libraries was verified via micro-capillary gel electrophoresis on a LabChip GX system (PerkinElmer, Waltham, MA). The libraries were quantified by fluorometry on a Qubit fluorometer (Life Technologies, Carlsbad, CA), and pooled in equimolar ratios. The library pool was quantified via qPCR with a Kapa Library Quant kit (Kapa Biosystems/Roche, Basel, Switzerland) on a QuantStudio 5 system (Applied Biosystems, Foster City, CA). The libraries were sequenced on a HiSeq 4000 sequencer (Illumina, San Diego, CA) with single-end 100 bp reads. 3′Tag-seq is a quick and efficient form of sequencing in which single-end sequencing is performed on the 3’ end, creating only an initial read of 80 or 90 base pairs. This creates low noise data that can easily be aligned to the existing, well annotated genome.

Reads were checked for quality using FastQC v0.11.9, then trimmed using bbduk, a function within bbmap (v37-50)^80^. Resulting reads were aligned to the *Aedes aegypti* LVP_AGWG-50 genome, indexed with an –sjdbOverhang 99 using STAR v2.7.2a^81,82^. Read files were then indexed using samtools v1.3.1^83^. Raw read data is available via the NCBI SRA database under the accession number PRJNA988225.

### Gene expression analysis

All statistical analyses were carried out using R Statistical Software (v.4.2.1)^84^. To observe similarities and groupings among samples, a principal component analysis and kmeans clustering analysis were performed.

Differential gene expression analysis was performed using edgeR^18^. To begin filtering the data set, genes with low expression (less than or equal to 1 CPM in more than 2 samples) were ignored. For a gene to be considered differentially expressed, a false discovery rate (FDR) of < 0.05 was used as a threshold. To characterize differential expression while considering time, a cubic regression spline curve with 3 degrees of freedom was used to assess expression trends across the time course with regard to treatment.

Co-expression network analysis was performed using the WGCNA package in R^60^. This analysis groups genes based on expression trends over time as well as their association with treatment.

Gene ontology enrichment analysis was performed using the R package TopGO^85^. All genes detectably expressed within this dataset were used as the background for this analysis. The TopGO fisher test was used to determine significance, using a *p* value cutoff of 0.05.

### Supplementary Information

Supplementary Information 1.

Supplementary Information 2.

## Data Availability

Raw data for this project can be found in the NCBI Sequence Read Archive with Bioproject accession number: PRJNA988225. https://www.ncbi.nlm.nih.gov/bioproject/?term=PRJNA988225.
