# Supplementary material for: Time-series analysis of transcriptomic changes due to permethrin exposure reveals that Aedes aegypti undergoes detoxification metabolism over 24 h
Source: Sci Rep. 2023 Oct 2;13:16564. doi: 10.1038/s41598-023-43676-9 (PMC10545687; doi:10.1038/s41598-023-43676-9)
Supplement: Supplementary file 1 — Supplementary Information 1. [file 41598_2023_43676_MOESM1_ESM.docx]

**Time-series analysis of transcriptomic changes due to permethrin exposure reveals that *Aedes aegypti* undergoes detoxification metabolism over 24 hours**

**Lindsey K. Mack^1^ and Geoffrey M. Attardo^1^***

**^1^Department of Entomology and Nematology, University of California, Davis, Davis, CA**

***Corresponding author: gmattardo@ucdavis.edu**

Supplementary file 1. File of all genes passing the 1 CPM in at least 2 samples filter. EdgeR statistics for all comparisons included. TC=time course (i.e. spline curves fit for comparison). TvsU=permethrin vs. acetone regardless of time.


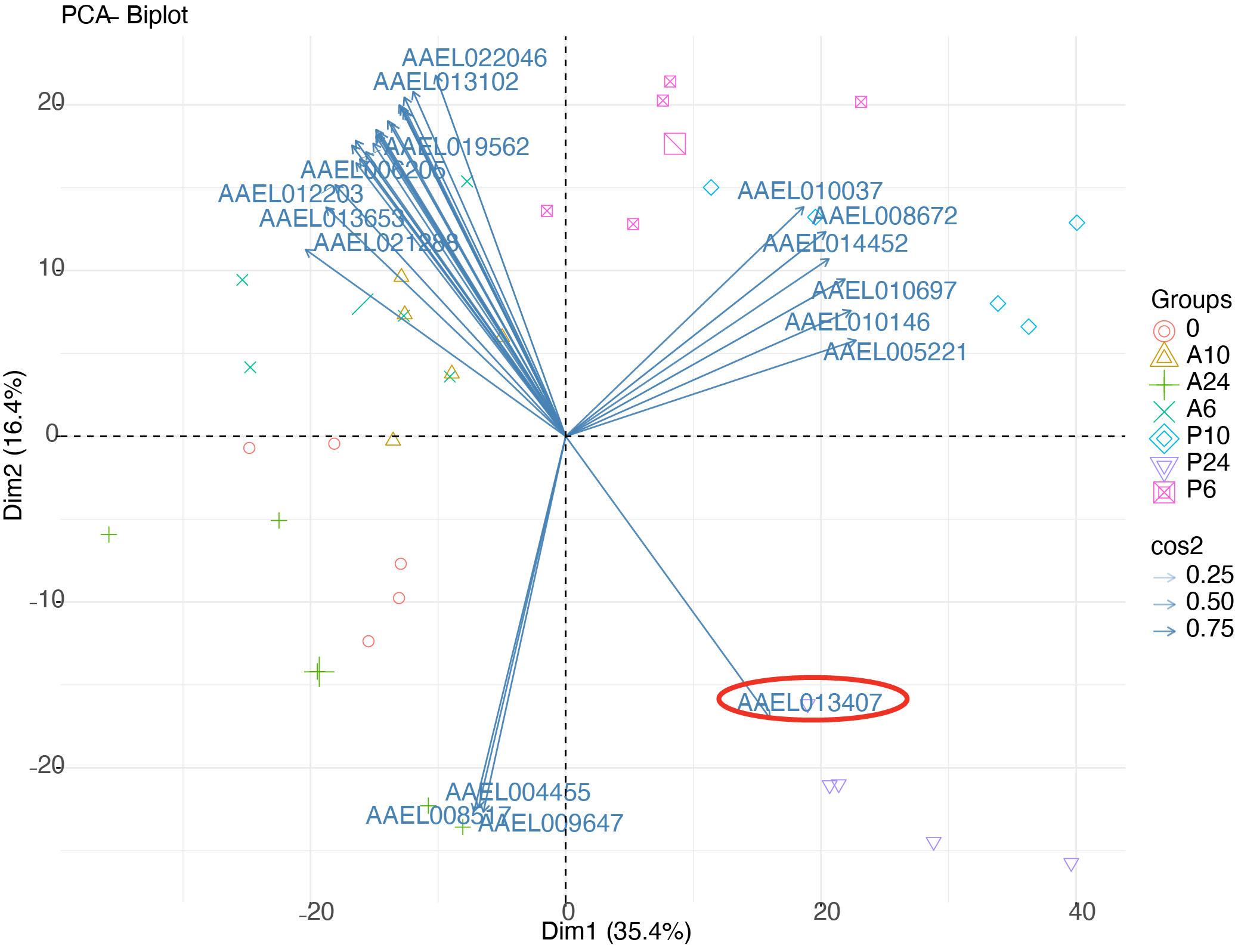


Supplementary Figure 1. Biplot of PCA in Fig. 1.


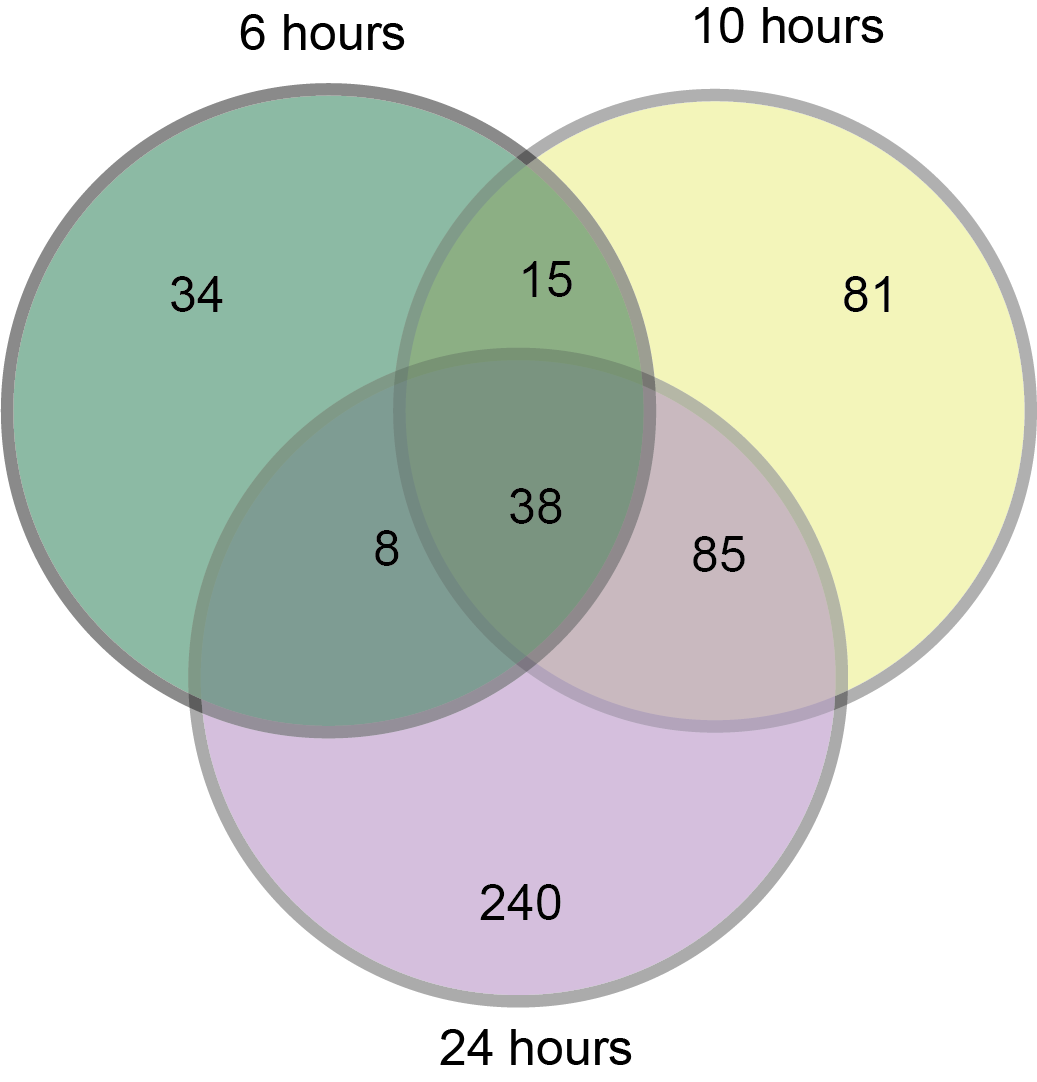


Supplementary Figure 2. Summary of pairwise gene expression overlap of upregulated genes, FDR < 0.05.

Supplementary Figure 3. Heat map from WGCNA analysis showing correlation with time and treatment. Module MEbrown is most associated with treatment.


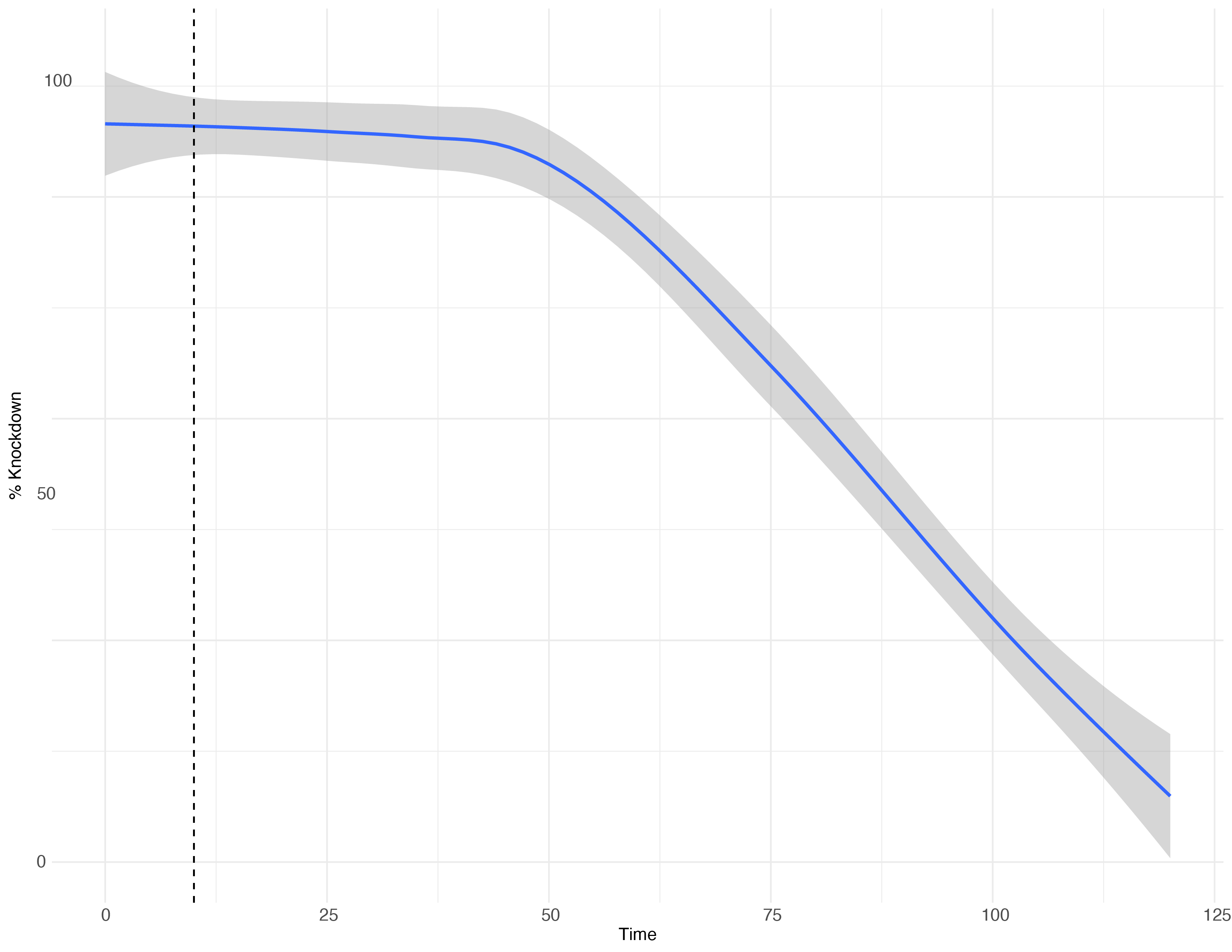


Supplementary Figure 4. Knockdown curve from CDC bottle bioassay. Dashed line indicated diagnostic time
